# Physiological and cognitive changes after treatments of cyclophosphamide, methotrexate, and fluorouracil: implications of the gut microbiome and depressive-like behavior

**DOI:** 10.3389/fnins.2023.1212791

**Published:** 2023-10-06

**Authors:** Christa Corley, Taylor McElroy, Bhavana Sridharan, Madison Trujillo, Pilar Simmons, Sangam Kandel, Delawrence J. Sykes, Michael S. Robeson, Antiño R. Allen

**Affiliations:** ^1^Division of Radiation Health, University of Arkansas for Medical Sciences, Little Rock, AR, United States; ^2^Department of Pharmaceutical Sciences, University of Arkansas for Medical Sciences, Little Rock, AR, United States; ^3^Department of Neurobiology and Developmental Sciences, University of Arkansas for Medical Sciences, Little Rock, AR, United States; ^4^Department of Bioinformatics, University of Arkansas for Medical Sciences, Little Rock, AR, United States; ^5^Department of Biology, Berry College, Mount Berry, GA, United States

**Keywords:** cyclophosphamide, methotrexate, 5-fluorouracil, hippocampus, amygdala, microbiome

## Abstract

**Introduction:**

Chemotherapy-induced cognitive impairment colloquially referred to as chemobrain is a poorly understood phenomenon affecting a highly variable proportion of patients with breast cancer. Here we investigate the association between anxiety and despair-like behaviors in mice treated with cyclophosphamide, methotrexate, and fluorouracil (CMF) along with host histological, proteomic, gene expression, and gut microbial responses.

**Methods:**

Forced swim and sociability tests were used to evaluate depression and despair-like behaviors. The tandem mass tag (TMT) proteomics approach was used to assess changes in the neural protein network of the amygdala and hippocampus. The composition of gut microbiota was assessed through 16S rRNA gene sequencing. Finally, quantitative reverse transcription polymerase chain reaction (qRT-PCR) was used to evaluate changes in intestinal gap junction markers.

**Results and discussion:**

We observed that CMF induced social and despair-like behavior in mice 96 hours following treatment. Proteomic analysis identified changes in various proteins related to progressive neurological disease, working memory deficit, primary anxiety disorder, and gene expression revealing increases in NMDA and AMPA receptors in both the hippocampus and the amygdala because of CMF treatment. These changes finally, we observed immediate changes in the microbial population after chemotherapy treatment, with a notable abundance of Muribaculaceae and Romboutsia which may contribute to changes seen in the gut.

## 1. Introduction

It is estimated that more than 4 million women are living in the US with a history of invasive breast cancer as of January 1, 2023, an additional 297,790 women will be newly diagnosed in 2023 (Siegel et al., 2023). With innovations in cancer therapy, survival rates have increased from 75% when diagnosed in the mid-70s to 90% as of 2017 (Siegel et al., 2023). With an increase in long-term survivorship, long-term symptoms that may follow the treatment of cancer have been identified. One of the original drug combinations used to treat breast cancer is CMF, which is a combination of cyclophosphamide, methotrexate, and fluorouracil. The use of CMF was first tested for use in treating aggressive breast cancer types in the US during the 1970s. One of the first studies assessing the cognitive function of patients with breast cancer following adjuvant chemotherapy, a study in which the majority of individuals received CMF, showed impaired mental functioning relative to test norms (Wieneke and Dienst Ev'elyn, 1995). A more recent study corroborates these findings and shows that patients treated with CMF performed worse in cognition functioning and had smaller gray matter than healthy individuals not diagnosed with breast cancer (Koppelmans et al., 2012a,b). Although taxanes and anthracyclines are now widely used in breast cancer treatments, studies of CMF effects are still valid because cyclophosphamide and fluorouracil are still used.

Common symptoms reported by patients treated with chemotherapy are cognitive decline, anxiety, and depression. Prior work by Schagen et al. (1995) revealed that patients treated with CMF and tamoxifen were 3.5 times more likely to experience cognitive dysfunction associated with attention, speed of information processing, motor speed, and visual memory compared to those patients not treated (controls). Furthermore, patients treated with high-dose chemotherapy were 8.2 times more likely to experience these conditions compared to low-dose patients. Similarly, a study in semirural South Africa also observed cognitive decline in women treated with CMF or fluorouracil, doxorubicin, and cyclophosphamide (FAC) (Keetile et al., 2021). A longitudinal study of patients with newly diagnosed breast cancer receiving anthracycline-based with taxane chemotherapy reports increased severity of cognitive impairment alongside anxiety and fatigue symptoms over a 15-month time frame (Ng et al., 2018). This evidence suggests that chemotherapy impairs cognition, including impairment of short-term memory, attention and concentration, long-term memory, processing speed, and overall executive functioning. Briones and Woods (2011), showed an association between a combination of CMF therapy and cognitive dysfunction and disruption of hippocampal neurogenesis, which decreases learning and memory. Similarly, Luczynski et al. (2016) showed volumetric and morphological changes in the amygdala, the integrative center for emotions, emotional behavior, and motivation under the hippocampus, disrupts social cognition and stress responsivity in germ-free (GF) C57/BL6 mice.

There have been various mechanisms explaining the increased rates of cognitive dysfunction and emotional changes such as neurotransmission disruption, systemic inflammation, blood brain barrier disruption and functional changes in the amygdala and hippocampus (Abu Hasan et al., 2022; Hu et al., 2022; Fleming et al., 2023) suggesting that, outside of increased general stress, there exist physiological mechanisms that increase these mood and cognitive symptoms during chemotherapy. Some of these cognitive and behavioral alterations may be mediated through the microbiome (Alexander et al., 2017; Loman et al., 2019; Grant et al., 2021; Sharvin et al., 2023). Recently many studies have shown a possible correlation between depressive symptoms and the gut microbiota. These microbiotas have been found to affect physiological functions, particularly metabolism, neurological and cognitive functions, inflammation, and immunity among others (Roy and Trinchieri, 2017; Peirce and Alviña, 2019; Ratsika et al., 2023). A healthy microbiota is comprised of a commensal community that works together to maintain health. Dysbiosis could lead to a representation of a pathogenic phenotype due to xenobiotics, therapeutic, treatments, diseases, etc. (Morais et al., 2021).

The microbiome gut–brain axis is a complex communication system found to establish a correlation between the altered gut microbiome and brain degeneration, which includes impairing many brain functions. Gastrointestinal microbes can produce a variety of molecules, including neurotransmitters, facilitating neurochemical communication between microbes and the host’s central, enteric, and autonomic nervous systems (Carabotti et al., 2015; Cryan et al., 2019). While the specific changes vary, chemotherapy-induced microbial disruption impairs the protective, immunomodulatory effects that the microbiome would normally provide to its host and increases the abundance of damaging products produced by pathogenic microbes. Several studies show intestinal microbial changes after chemotherapy treatment (Wei et al., 2021). The most common change in these studies is a decrease in microbial diversity (Ma et al., 2019; Wei et al., 2021; Huang et al., 2022; Wu et al., 2022).

The goal of this study was to investigate changes in the microbiome and its association with cognitive impairments controlled by the hippocampus and anxiety-like behaviors controlled by the amygdala. Here, we investigate the associations between depressive symptoms and memory and the microbiome.

## 2. Methods

### 2.1. Animals

Three-month-old female C57BL6/J mice (Jackson Laboratory) were group housed 4–5 per cage under a constant 12-h light:12-h dark; 6 am/6 pm cycle in a climate-controlled environment. Food and water were provided *ad libitum*. Mice in the same treatment groups were housed together. All procedures were approved by the Institutional Animal Care and Use Committee at the University of Arkansas for Medical Sciences (UAMS; Little Rock, Arkansas).

### 2.2. Chemotherapy

**Figure 1:**
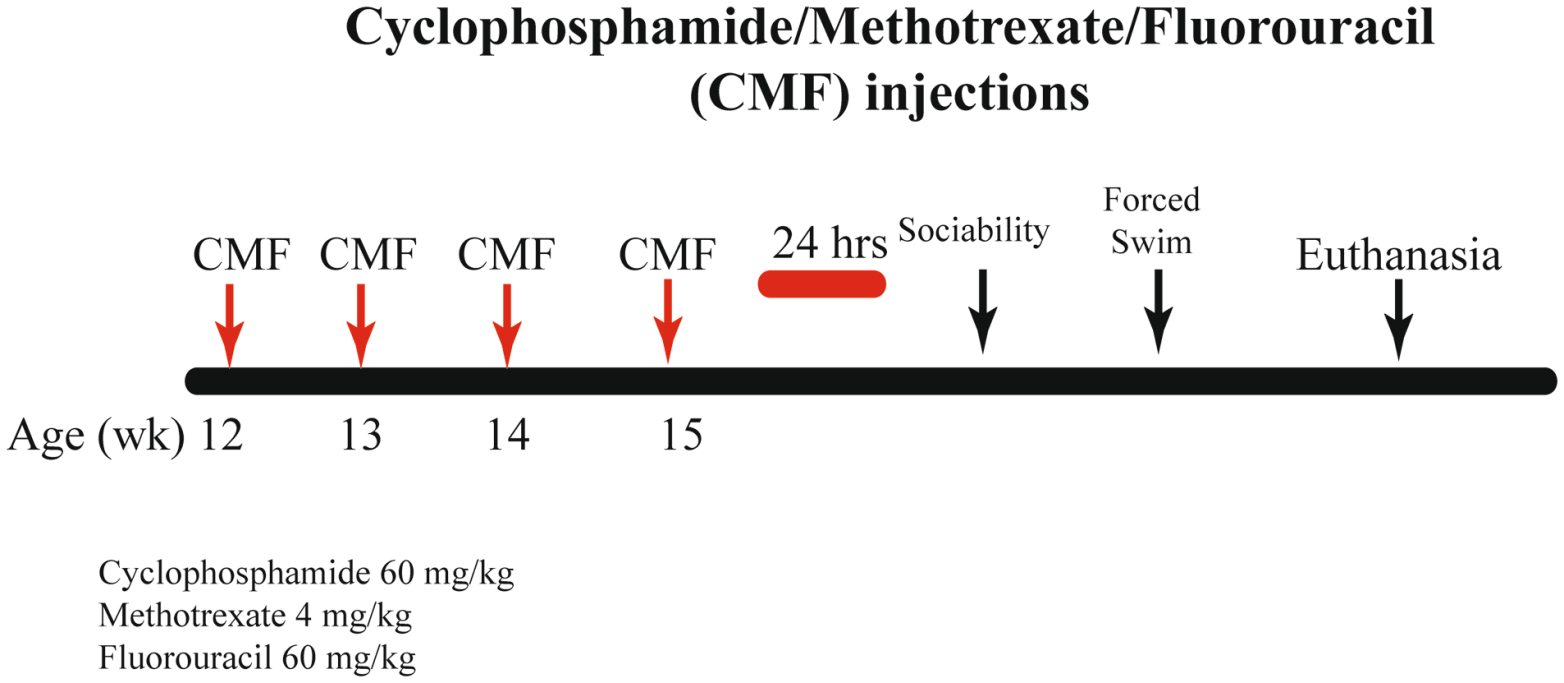
Schematic timeline for study procedure. Twelve-week-old C57BL/6J Female mice received 4 weekly intraperitoneal injections of either saline or CMF. Behavior testing was initiated 24 h after the last injections.

Cyclophosphamide, methotrexate, and fluorouracil were purchased from the UAMS Inpatient Pharmacy. For 4 weeks, mice received weekly intraperitoneal (IP) injections on days 1, 8, 15, and 22. The control group received saline (0.9% sodium chloride) and the CMF group received 60 mg/kg cyclophosphamide, 4 mg/kg methotrexate, and 60 mg/kg fluorouracil (Anderson et al., 2020). The drugs were combined prior to injection then administrated. Chemotherapy dosages were translated from standard dosages for human to be clinically relevant. These dosages were translated by normalizing body surface area. Drugs were diluted with sterile saline and stored per the manufacturer’s instructions. Drugs were mixed immediately before injections. A schematic of the experimental timeline can be found in Figure 1.

### 2.3. Behavioral scheme and analysis

Mice were handled before the onset of behavioral testing to acclimate them to handling. Prior to each day of testing, mice were acclimated to the testing room in their home cage for 1 h. Behavior testing began 24 h after final injection.

#### 2.3.1. Forced swim test

For the Forced swim test (FST), mice were observed for their mobility after test injections. A cylindrical tank (30 cm height x 20 cm diameter) made of Plexiglas was filled with room-temperature water and maintained approximately 24°C ± 2°. The mice were gently placed in the water while being closely monitored. Recording sessions lasted approximately 7 min. After recording, the mouse was placed back into home cages (Can et al., 2012). Data were analyzed by comparing time spent immobile vs. mobile (*n* = 10 per treatment group). Methods can be referenced to Can et al. (2012).

#### 2.3.2. Sociability test

**Figure 2:**
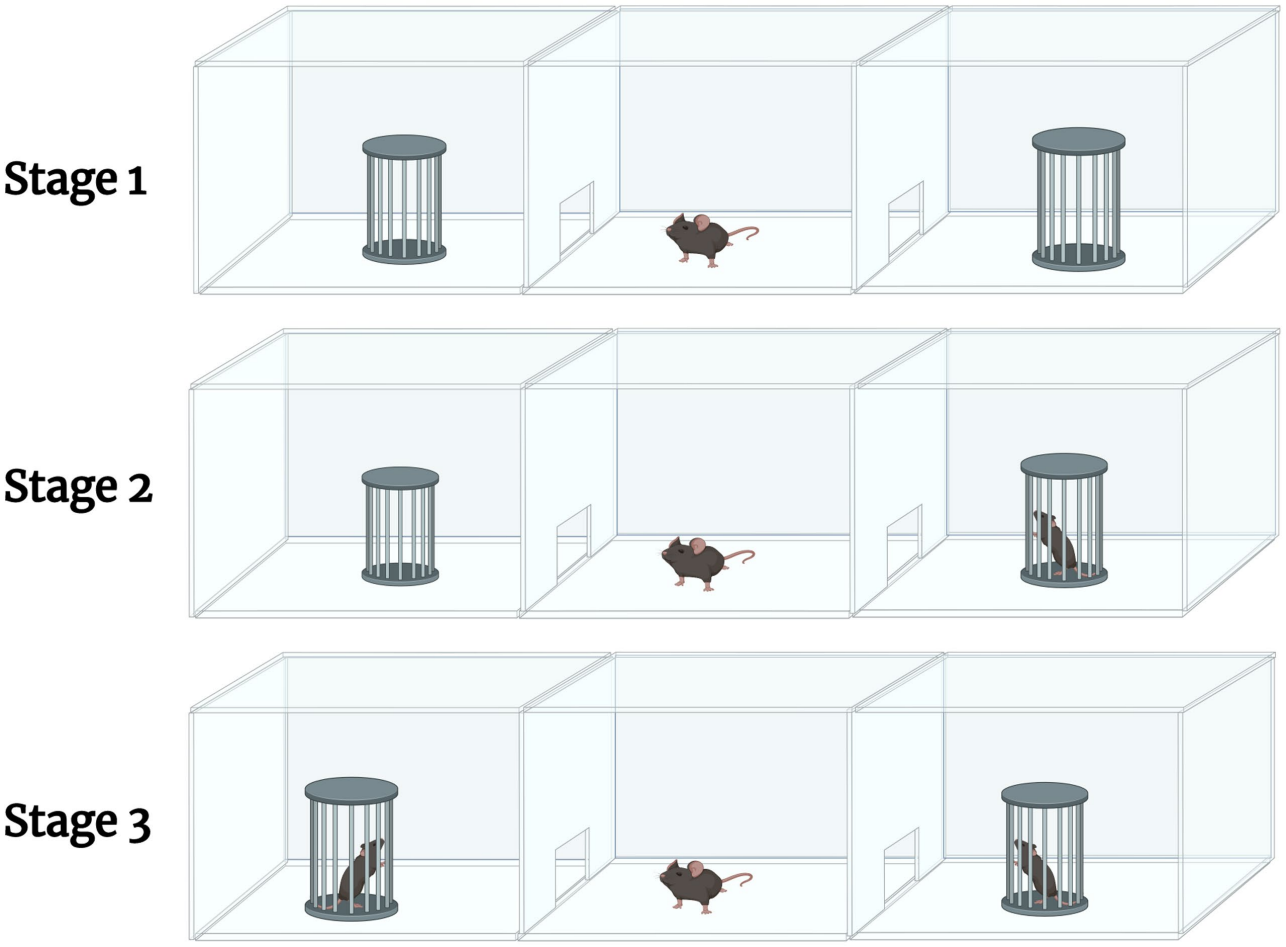
A graphical description of the 3-chamber sociability test. The test involves 3 stages. Stage 1 allows for the habituation of the test mouse, where it is allowed to explore the apparatus. Stage 2 involves introducing an unfamiliar mouse (Stranger 1) in one of the empty chambers. The test mouse is allowed to familiarize itself with Stranger 1. During Stage 3, another unfamiliar mouse (Stranger 2) is introduced into the opposite, previously empty chamber. The test mouse is allowed to familiarize itself with Stranger 2. Created in Biorender.com.

Three Chamber Sociability test (Yang et al., 2011) consisted of a polycarbonate box with removable partitions separating the box into 3 chambers. The partitions had openings that allowed the mouse to move freely from chamber to chamber. The test mouse was placed in the middle chamber with the dividers open to allow access to all 3 chambers. After a 10-min habituation period, an unfamiliar mouse of the same strain, sex, and age (Stranger 1) was placed inside a small wire cage in one of the side chambers. The mouse was allowed to familiarize itself with Stranger 1 for 10 min. While Stranger 1 remained in its wire cage on one side of the apparatus, a new unfamiliar mouse of the same strain, sex, and age (Stranger 2) was placed in a previously empty wire cage in the chamber on the opposite side of the apparatus. The mouse was allowed to familiarize itself with Stranger 2 for 10 min. Data were analyzed by comparing the percent of time spent with Stranger 1 vs. Stranger 2. A graphical depiction can be found in Figure 2 (*n* = 10 per treatment group). Methods can be reference from Semple et al. (2012).

#### 2.3.3. Sample collection and tissue preparation

Mice were euthanized by cervical dislocation at 96 h (*n* = 10 per treatment) after the last chemotherapy injection. Brains were removed, dissected in cold phosphate-buffered saline, divided longitudinally by hemisphere, flash-frozen in liquid nitrogen, and stored at −80°C. The whole brain was flash-frozen in 2-methylbutane and stored at −80°C. The hippocampus and amygdala were extracted from coronal sections of the brain using a 0.2 mm tissue punch.

#### 2.3.4. Proteomics analysis

Total protein from each tissue sample was reduced, alkylated, and purified by chloroform/methanol extraction prior to digestion with sequencing grade modified porcine trypsin (Promega). Tryptic peptides were labeled using tandem mass tag isobaric labeling reagents (Thermo) following the manufacturer’s instructions and combined into one 16-plex TMTpro sample group. The labeled peptide multiplex was separated into 46 fractions on a 100 × 1.0 mm Acquity BEH C18 column (Waters) using an UltiMate 3,000 UHPLC system (Thermo) with a 50 min gradient from 99:1 to 60:40 buffer A:B ratio under basic pH conditions, and then consolidated into 18 super-fractions. Each super-fraction was then further separated by reverse phase XSelect CSH C18 2.5 um resin (Waters) on an in-line 150 × 0.075 mm column using an UltiMate 3,000 RSLCnano system (Thermo). Peptides were eluted using a 70 min gradient from 98:2 to 60:40 buffer A:B ratio. Eluted peptides were ionized by electrospray (2.4 kV) followed by mass spectrometric analysis on an Orbitrap Eclipse Tribrid mass spectrometer (Thermo) using multi-notch MS3 parameters. MS data were acquired using the FTMS analyzer in top-speed profile mode at a resolution of 120,000 over a range of 375 to 1,500 m/z. Following CID activation with normalized collision energy of 35.0, MS/MS data were acquired using the ion trap analyzer in centroid mode and normal mass range. Using synchronous precursor selection, up to 10 MS/MS precursors were selected for HCD activation with normalized collision energy of 65.0, followed by acquisition of MS3 reporter ion data using the FTMS analyzer in profile mode at a resolution of 50,000 over a range of 100–500 m/z.

Buffer A = 0.1% formic acid, 0.5% acetonitrile.

Buffer B = 0.1% formic acid, 99.9% acetonitrile.

Both buffers adjusted to pH 10 with ammonium hydroxide for offline separation.

Methods were performed by UAMS Proteomic Core (*n* = 3 per treatment group).

#### 2.3.5. RNA extraction and qRT-PCR

Total RNA was extracted from the hippocampus, amygdala, and jejunum with the PureLink RNA Mini Kit (ThermoFisher Scientific). Changes that were made in the lysis step are as follows: QIAzol (QIAGEN) was used as a lysis buffer, then samples were homogenized with a Bio-Gen Series PRO200 homogenizer. Chloroform (200 μL) was added to each sample and inverted until the sample turned into a milky pink solution. After resting for 5 min, samples were centrifuged at 12,000 G for 15 min. The supernatant was collected while avoiding the interphase layer. Lastly, 70% ethanol was added in equal volumes of supernatant. Wash and elution steps were followed per manufacturer protocol.

RNA quality and quantity were assessed on a Nanodrop 2000 spectrophotometer (ThermoFisher Scientific). cDNA was synthesized with random primers and a High-Capacity cDNA Reverse Transcription Kit (ThermoFisher Scientific) according to the manufacturer’s protocol. The levels of gene transcripts were determined by qRT-PCR with TaqMan Gene Expression Assays (ThermoFisher Scientific), according to the manufacturer’s protocol. In all cases, 18S ribosomal RNA (18S rRNA) was used as an internal reference gene, and fold changes were calculated with the 2^−ΔΔCt^ method. Measurements were performed in duplicate. TaqMan Assays used are listed in Supplementary Table S1 (*n* = 7 per treatment group; hippocampus and amygdala) (*n* = 8 per treatment group; jejunum samples).

#### 2.3.6. Analyzing 16S rRNA gene sequences

Mouse fecal pellets were sent to RTL Genomics (Lubbock, Texas) for DNA extraction, amplification, and sequencing of the V3-V4 small ribosomal subunit (16S rRNA) hypervariable region, using the following primers: 5’-CCTACGGGNGGCWGCAG-3′, 5’-GACTACHVGGGTATCTAATCC-3′ (Klindworth et al., 2013). MIMARKS (Yilmaz et al., 2011) compliant sequencing data is available via the GenBank SRA under BioProject PRJNA948457.

Microbiome analyses were performed with QIIME 2 (Bolyen et al., 2019) version 2021.11. were demultiplexed and the primers trimmed. Any references prefixed with q2- are QIIME 2 plugins. Demultiplexed FASTQ files were imported in QIIME2 as QIIME Zipped Artifacts (qza) using q2-import and visualized using q2-demux summarize via QIIME2. Amplicon Sequence Variants (ASVs)/Exact Sequence Variants (ESVs) (Callahan et al., 2016b) were generated from forward reads with DADA2 (Callahan et al., 2016a) via q2-DADA2 plugin.

Taxonomy was assigned to ASVs with Naïve Bayes classifier trained on SSU SILVA NR99 reference database (Pruesse et al., 2007; Quast et al., 2013) q2-feature-classifier classify-sklearn plugin (Bokulich et al., 2018) RESCRIPt (Robeson et al., 2021) was used to curate the SILVA NR99 v138.1 reference database. ASVs that were classified as ‘Unclassified’, ‘Mitochondria’, ‘Chloroplast’, ‘Eukaryotes’, and those not having at least phylum level classification were removed. The quality of the sequences was evaluated with q2-quality-control evaluate-seqs plugin by comparing the feature sequences to the curated SILVA reference, any sequences that did not have at least either a 90% identity or query coverage were removed.

Data was rarefied at 4,471 reads per sample. Alpha diversity metrics were estimated for observed taxa, Shannon Index, and Faith’s Phylogenetic Diversity (PD). Beta diversity was estimated with UniFrac (weighted and unweighted) (Lozupone and Knight, 2005) and Bray–Curtis dissimilarity and Jaccard with q2-diversity.

### 2.4. Assessment of villus height and crypt depth

Segments of proximal jejunum were obtained, fixed, embedded so that four transverse sections were obtained per specimen, cut at 5 μm, and stained with Hematoxylin and eosin (H&E). H&E-stained slides were used for villi length and crypt depth determination. Each stained section was examined for histopathological abnormalities on a microscope supported with a digital camera. Images were captured at 4x magnification for villus measurements and 10x magnification for crypt measurements. An average of 12 villi and crypts were, respectively, analyzed for villous height and crypt depth measurements per animal. The villus height was measured from the tip to the villus-crypt junction and the crypt depth from the base of the villus to the mucosa using the image analysis software ImageJ (v.1.53; National Institutes of Health, Bethesda, MD) (*n* = 5 per treatment group). Methods can be reference to Banerjee et al. (2019).

### 2.5. Statistical analysis

Data were expressed as means ±SEM. Comparisons between means were carried out using one-way ANOVA when analyzing the Three Chamber Sociability. Unpaired *t*-test were performed on forced swim test and PCR results. Statistical analysis was performed using GraphPad Prism software version 9 (San Diego, CA, USA); a probability level of less than 0.05 was accepted as statistically significant. Statistical analysis for QIIME 2 analysis has been identified in methods.

## 3. Results

### 3.1. Animals

**Figure 3:**
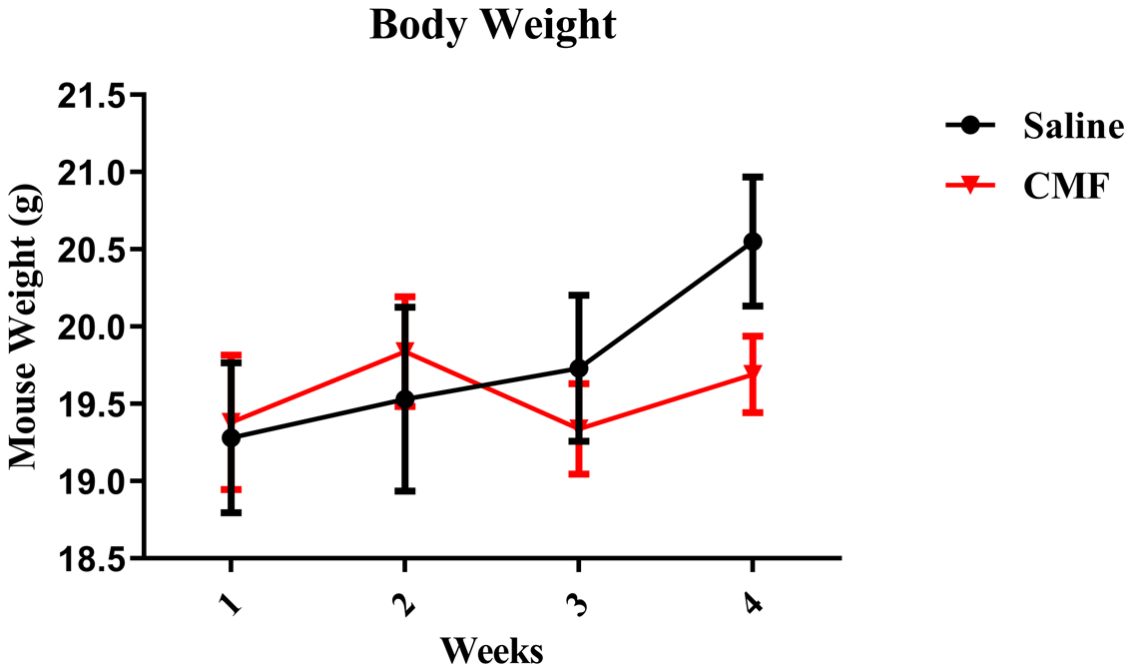
Animal weight. The weights of animals are shown as grams. There were no changes in body weights during chemotherapy (*N* = 10 per treatment). Error bars represent mean ± SEM.

Body Weights were tracked during the injection period. There were no significant changes identified in weight between groups [*F*_(3, 54)_ = 1.249, *p* = 0.3012; Figure 3].

### 3.2. Force swim test

**Figure 4:**
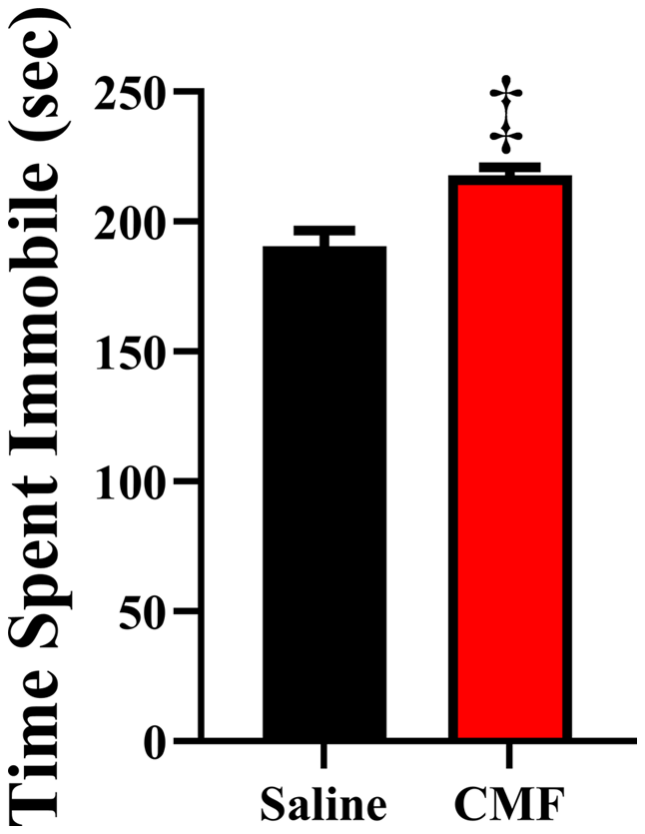
The forced swim test measured despair/depressive-like behaviors. Mice treated with CMF showed significantly more time being immobile when compared with mice treated with saline (*N* = 10 per treatment). *p < 0.05; † *p* < 0.01; ‡*p* < 0.001; ⁑*p* < 0.0001.

FST showed that 30 days after the last treatment, mice treated with CMF spend significantly more time being immobile than mice that were treated with saline (*t* = 2.230, df = 18, *P* = 0.0387; Figure 4).

### 3.3. Sociability

**Figure 5:**
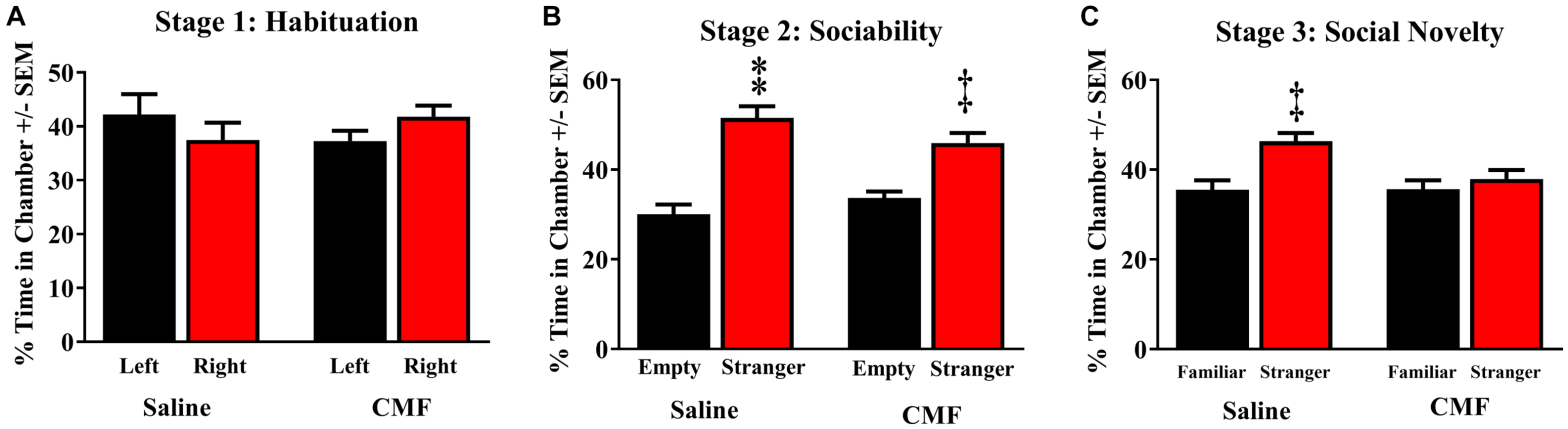
Social memory test. **(A)** Animals of the 3 cohorts displayed no significant bias of chamber exploration [*F*_(3, 36)_ = 0.8908; *P* = 0.4552]. **(B)** All cohorts displayed normal sociability by spending significantly more time with Stranger 1 when the other chamber was empty [*F*_(3, 36)_ = 21.96; *P* < 0.0001; CMF, *P* < 0.0006]. **(C)** Saline-treated mice were able to discriminate between Stranger 1 and Stranger 2 when both mice were present, whereas mice treated CMF failed to distinguish between them *r* [*F*_(3, 36)_ = 6.584; *P* = 0.0012; Holm-Sidak stimulus vs. empty; saline *P* = 0.0010; CMF *P* = 0.6587] (*n* = 10 per treatment). *p < 0.05; †*p* < 0.01; ‡*p* < 0.001; ⁑*p* < 0.0001.

The 3-chamber sociability test suggested social impairments in mice treated with CMF. All mice demonstrated the ability to properly habituate by spending nearly equal amounts of time in each outer chamber in Stage 1 [*F*_(3, 36)_ = 0.8908; *P* = 0.4552; Figure 5A]. Both treatment groups demonstrated the ability to socialize properly by spending significantly more time in the chamber with Stranger 1 in it as opposed to the opposite empty chamber in Stage 2 [*F*_(3, 36)_ = 21.96; *P* < 0.001]. With the Holm-Sidak method for multiple comparisons, we compared the chambers stimulus vs. empty (saline, *P* < 0.0001; CMF, *P* < 0.0006; Figure 5B). In Stage 3, mice treated with CMF demonstrated the inability to discriminate between the Familiar and Stranger, while the saline group successfully discriminated by spending more time with the stranger mouse than with the familiar [*F*_(3, 36)_ = 6.584; *P* = 0.0012; Holm-Sidak stimulus vs. empty; saline *P* = 0.0010; CMF *P* = 0.6587; Figure 5C].

### 3.4. Proteomics

#### 3.4.1. Ingenuity pathway analysis

**Figure 6:**
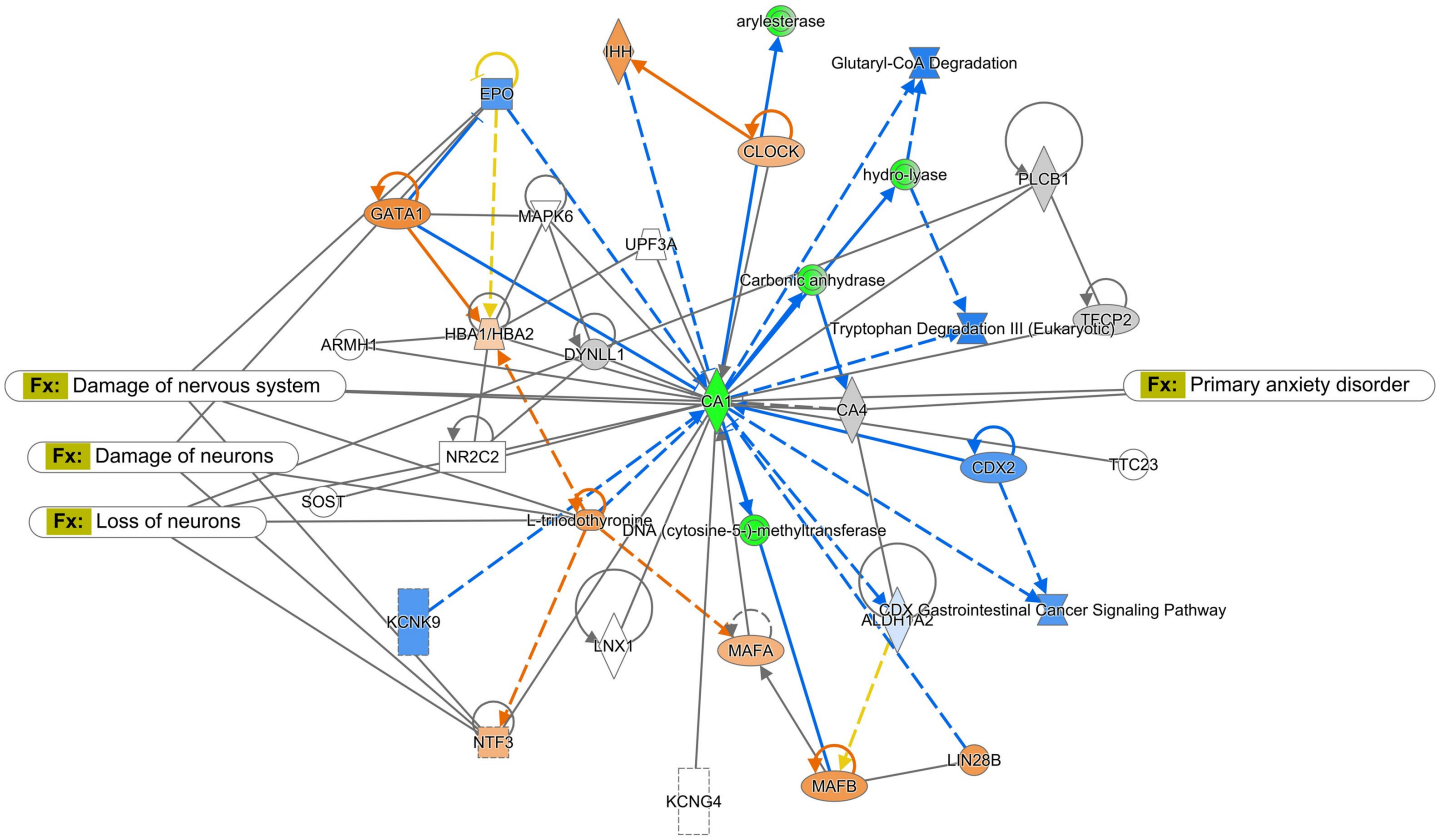
Ingenuity pathway analysis for the amygdala. Graphic presentation for the amygdala as identified by Ingenuity Pathway Analysis (IPA) for saline-treated and CMF-treated. The network is overlayed with the disease and function tool to display the 4 key molecules involved with neurological disorders. The functions associated with the network are Neurological Disease, Cellular Development, Organismal Injury and Abnormalities, and Hereditary Disorders. The node color indicates expression value and color intensity indicates the degree of upregulation (red) or downregulation (green). Gray nodes are dataset molecules that were not significantly expressed and therefore did not pass the IPA analysis cutoff. Uncolored nodes were not part of our dataset but were incorporated into the pathway based on evidence stored in the Ingenuity Knowledge Base. Known direct and indirect interactions between network proteins, as well as the direction of the interaction, are indicated by arrows or blocked lines.

**Figure 7:**
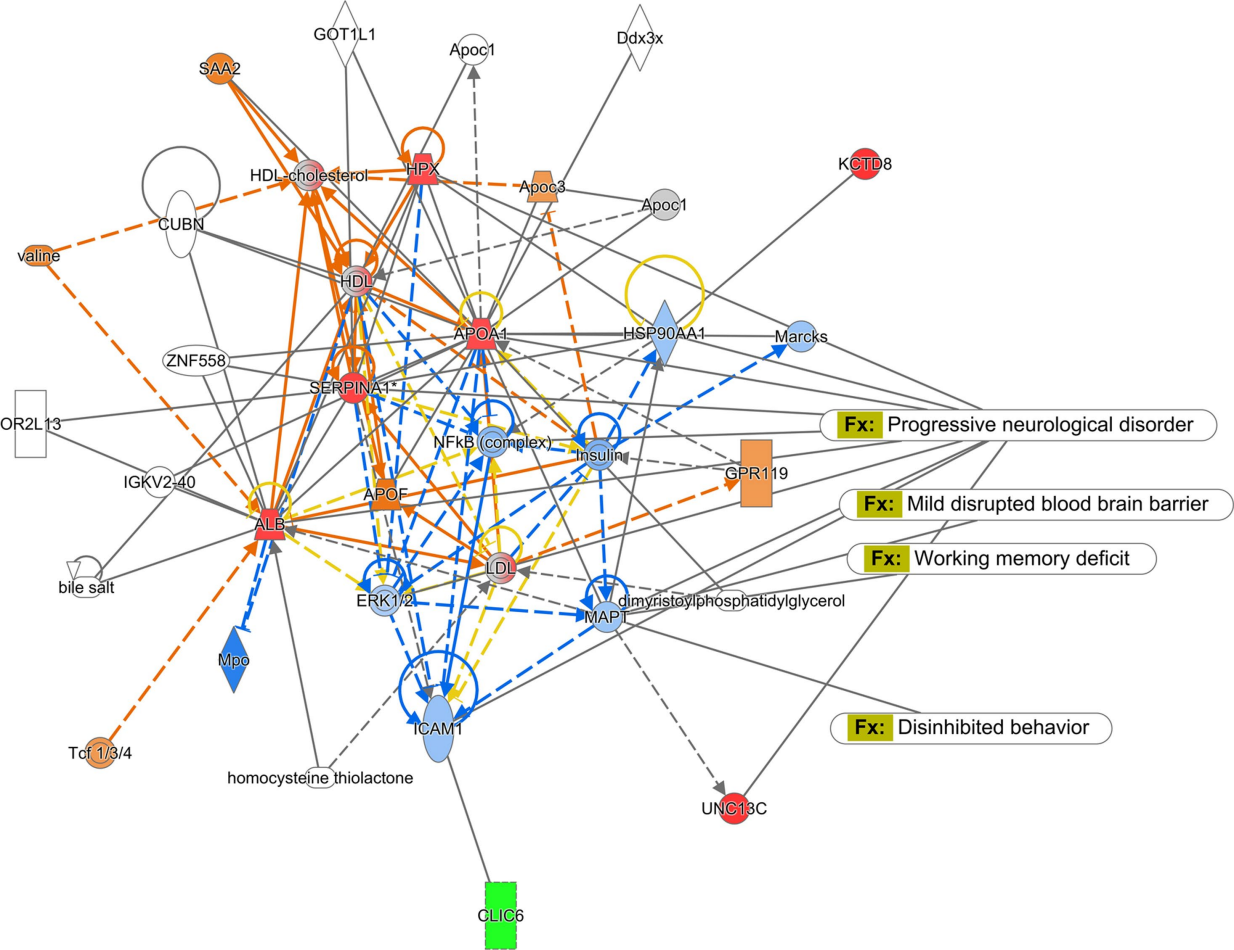
Ingenuity pathway analysis for hippocampus. Graphic presentation for the hippocampus as identified by Ingenuity Pathway Analysis (IPA) for saline-treated and CMF-treated. The functions associated with the network are Cellular Development and Function, Organ Morphology, Neurological Disease. The network is overlayed with the disease and function tool to display the 4 key molecules involved with neurological disorders. The node color indicates expression value and color intensity indicates the degree of upregulation (red) or downregulation (green). Gray nodes are dataset molecules that were not significantly expressed and therefore did not pass the IPA analysis cutoff. Uncolored nodes were not part of our dataset but were incorporated into the pathway based on evidence stored in the Ingenuity Knowledge Base. Known direct and indirect interactions between network proteins, as well as the direction of the interaction, are indicated by arrows or blocked lines.

The Ingenuity Pathway Analysis (Qiagen) was performed for protein expression in potential pathways and networks associated with the saline-treated vs. the CMF-treated mice. We performed this analysis on both the amygdala and hippocampus tissues. From the raw data, 5,165 molecules were found in the amygdala and 2,407 proteins in the hippocampus. Both sets of data were filtered for proteins with a 1.5-fold change and a value of *p* of 0.55. We used data from differentially expressed proteins and focused on the top network with the highest z-score and involved neurological disease. In the amygdala, we used the disease and function overlay tool to investigate protein association within the network. Protein associations within the network include damage to the nervous system, damage to neurons, loss of neurons, and primary anxiety disorder. The common protein among these disorders and functions is neurotrophin 3 (NTF3) and carbonic anhydrase 1 (CA1). We applied the same overlay in the hippocampus. Proteins within this network are predicted to be associated with progressive neurological disorder, mild disrupted blood–brain barrier, working memory deficit, and disinhibited behavior. The common protein among these overlays is microtubule associate protein tau (MAPT). Protein networks can be found in Figures 6, 7. Complete list of proteins can be found in the Supplementary material Protein identified in each network can be found in Supplementary Tables S2, S3.

### 3.5. Modification in expression of AMPA and NMDA receptors

**Figure 8:**
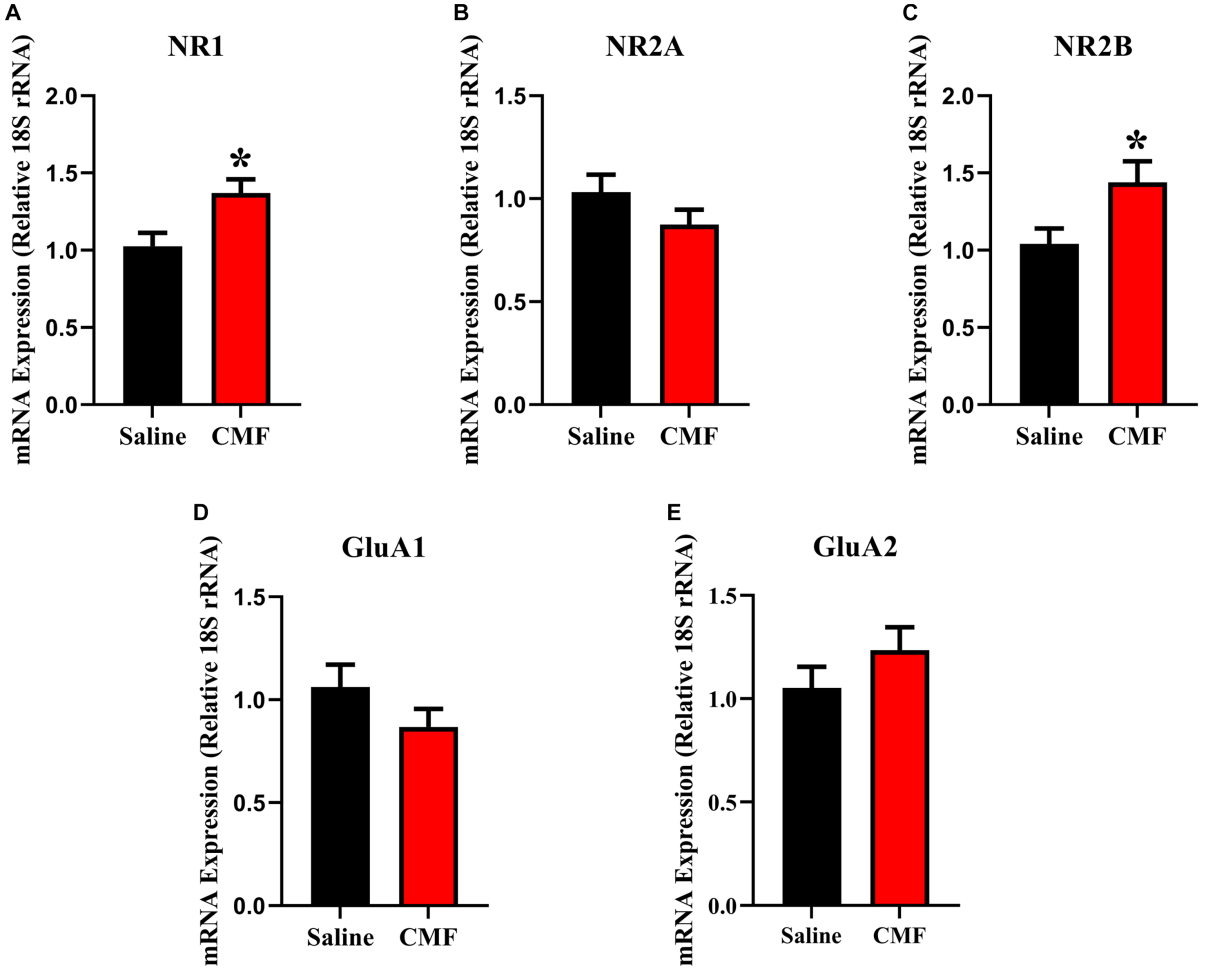
In the amygdala, CMF induces early alterations in NMDA receptor gene expression. **(A)** The mRNA levels of NR1 and **(C)** NR2B were significantly increased in CMF-treated mice compared to saline-treated mice, but not significant in **(B)** NR2A, **(D)** GluA1, and **(E)** Glu2A. Data are presented as fold change in gene expression relative to saline as control (*N* = 8 per treatment). *p < 0.05; †*p* < 0.01; ‡*p* < 0.001; ⁑*p* < 0.0001.

**Figure 9:**
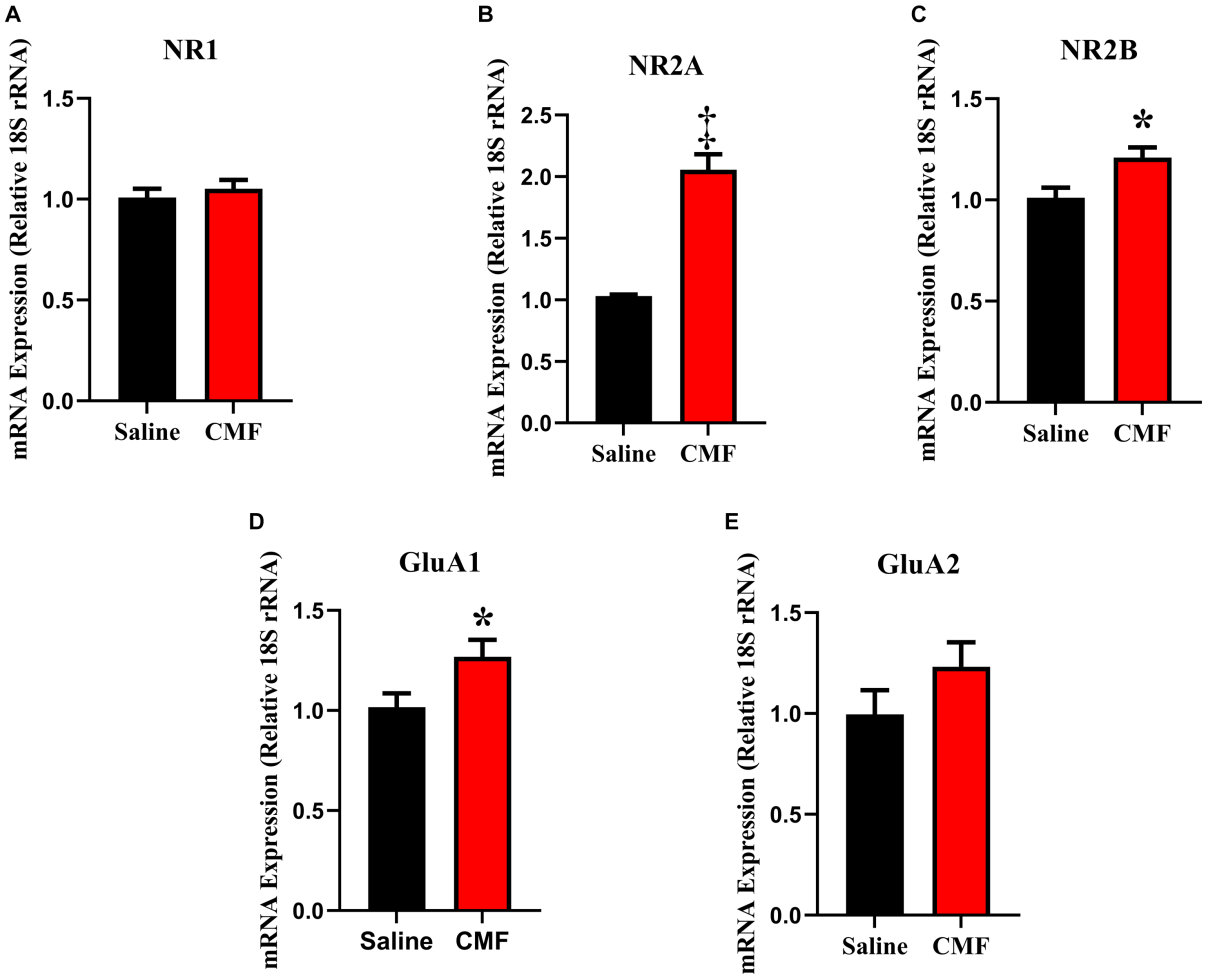
In the hippocampus, CMF induces early alterations in NMDA receptor gene expression. In the hippocampus, CMF induces early alterations in NMDA receptor gene expression. **(A)** The mRNA levels of NR1 were not significantly different; **(B)** NR2A and **(C)** NR2B expression was significantly increased in the CMF-treated group. **(D)** Glutamate receptor GluA1 was also increased in the CMF-treated group, but not **(E)** GluA2. Data are presented as fold change in gene expression relative to saline as control (*N* = 8 per treatment).*p < 0.05; †*p* < 0.01; ‡*p* < 0.001; ⁑*p* < 0.0001.

In this study, we examined the mRNA expression of N-methyl-D-aspartate (NMDA) subunits, NR1, NR2A, and NR2B, and a-amino-3-hydroxy-5-methyl-4-isoxazole propionic acid (AMPA) subunits, GluAl and GluA2 in the amygdala and hippocampus of mice treated with saline and CMF. We found an upregulation of NR1 (*t* = 2.829, df = 16.98, *P* = 0.0116; Figure 8A) and NR2B (*t* = 2.365, df = 16.03, *P* = 0.0310; Figure 8B) in the amygdala of the CMF- treated mice. In the hippocampus, we found an up regulation of NR2A (*t* = 7.444, df = 9.717, *P* < 0.0001; Figure 9A), NR2B (*t* = 2.832, df = 16.95, *P* = 0.0115; Figure 9B), and GluA1 (*t* = 2.291, df = 16.57, *P* = 0.0353; Figure 9C).

### 3.6. Analyzing changes in microbial composition using QIIME2

#### 3.6.1. Taxonomy classification

**Figure 10:**
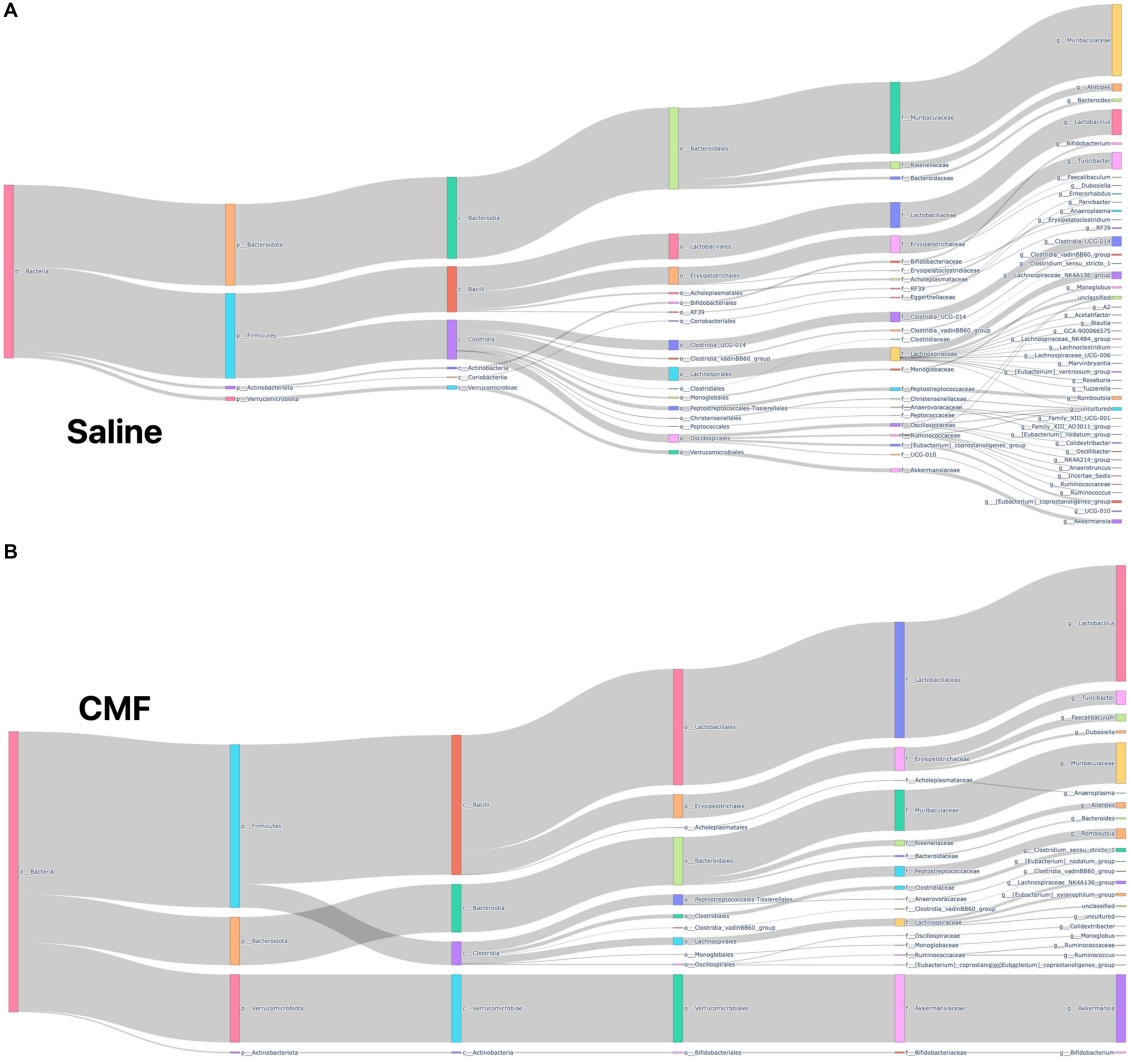
Sankey plots. **(A)** Saline **(B)** CMF Depiction of microbial population within our sample with we see visual representation in the changes of species when comparing CMF to saline. Through Aldex2 and ANCOM, two taxa were found to be differentially expressed between the two groups.

The microbiome composition of the saline-treated mice and chemotherapy-treated mice was depicted using Sankey plots (Figures 10A,B). Microbiome composition of the mice treated with CMF after 96 h was different from those of mice that were treated with saline. Visually, we see a shift in microbial populations. Statistically, on the phylum level, *Firmicutes* and *Bacteriodota* were differential abundant which was verified using Aldex2 and ANCOM.

#### 3.6.2. Alpha diversity of the microbial population

**Figure 11:**
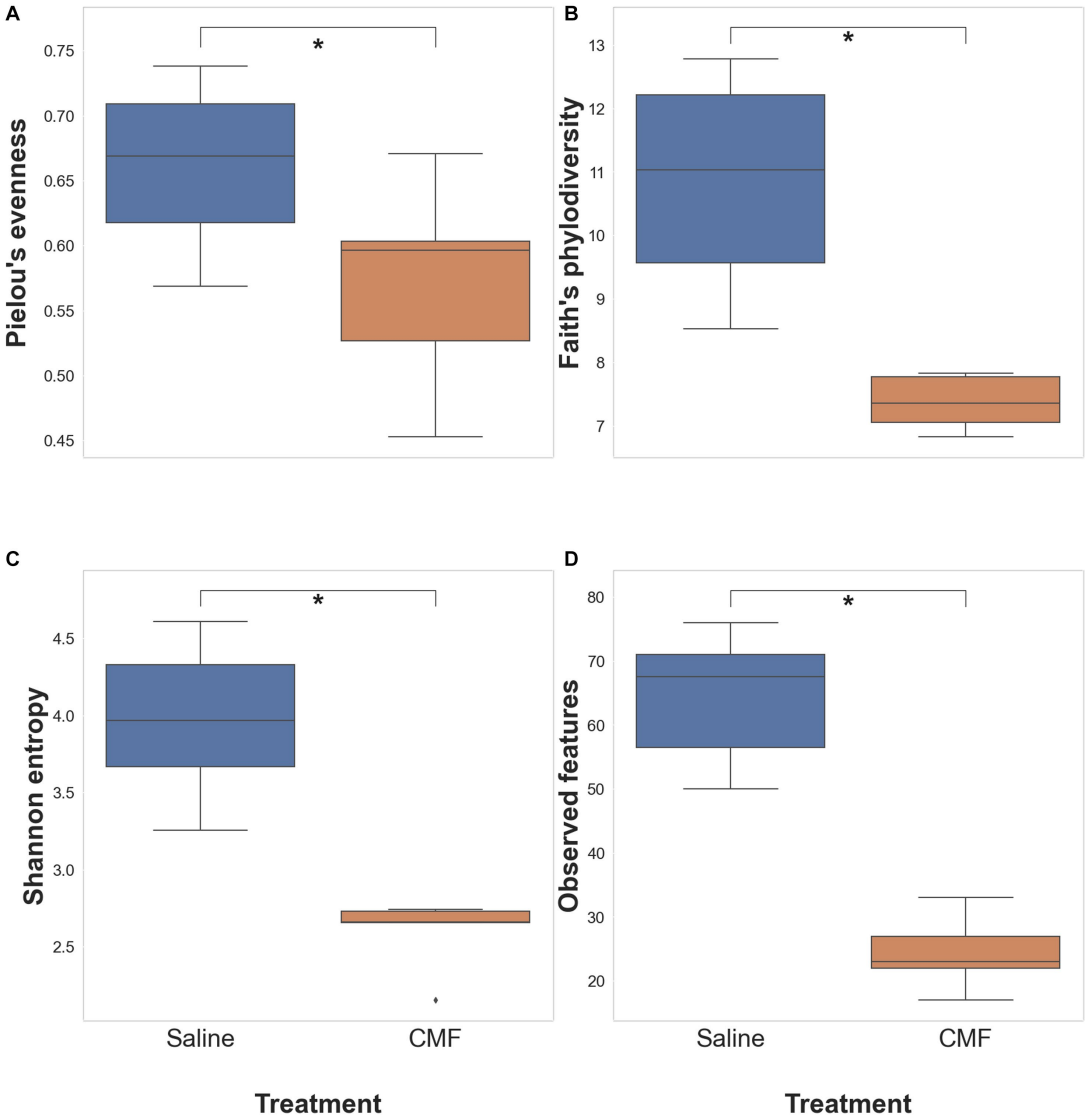
Alpha diversity analysis. Differences within the populations with **(A)** Pielous’s Evenness (*p* = 0.0678), **(B)** Faith’s PD (*p = 0.00617), **(C)** Shannon’s Entropy (*p = 0.00617), and **(D)** Observed taxa (*p = 0.00617) (*n* = 6 saline; 5 CMF).

The significance of each diversity metric was tested with the Pairwise Kruskal-Wallis test between saline-treated and CMF-treated mice. Kruskal-Wallis (pairwise) did not identify any significant differences in Pielou’s evenness (Figure 11A), significant differences were observed with Shannon Entropy (Figure 11B), and Faith’s PD (Figure 11C) and, observed taxa (Figure 11D).

#### 3.6.3. Beta diversity of the microbial population

**Figure 12:**
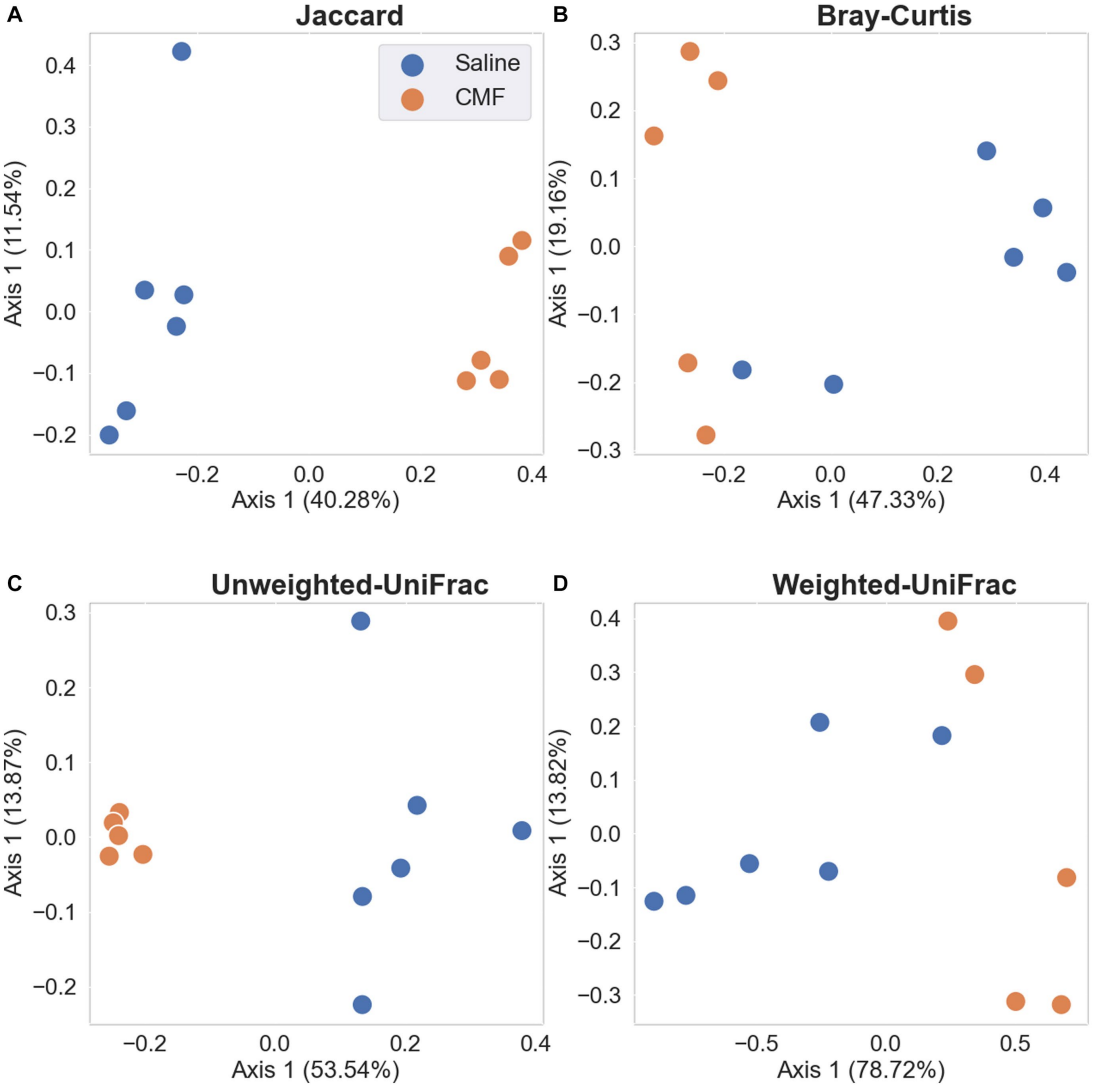
PCA plots with Emperor. Depiction of the Beta Diversity of the microbial populations between saline-treated vs. CMF-treated. **(A)** Jaccard Emperor, **(B)** Unweighted Unifrac, **(C)** Bray-Curtis Emperor, **(D)** Weighted Unifrac. We see separation when abundance is not considered in the Jaccard and Weighted Unifrac charts.

Differences between sample diversity were estimated with Jaccard distance (Figure 12A), Unweighted UniFrac distance (Figure 12B), Bray–Curtis dissimilarity matrix (Figure 12C), and Weighted UniFrac distance (Figure 12D). Statistical analysis identifying differential abundance using Aldex2 identified two significantly different features between the two groups found in the Phylum, Bacteroidota, and Firmicutes (*p* = 0.0398 and *p* = 0.0474 respectively) using the Benjamini-Hochberg corrected *p* value of Welch’s *t*-test, following an ANCOM-BC analysis which identified Firmicutes rejected the null hypothesis.

### 3.7. CMF treatment is associated with intestinal structural derangement

**Figure 13:**
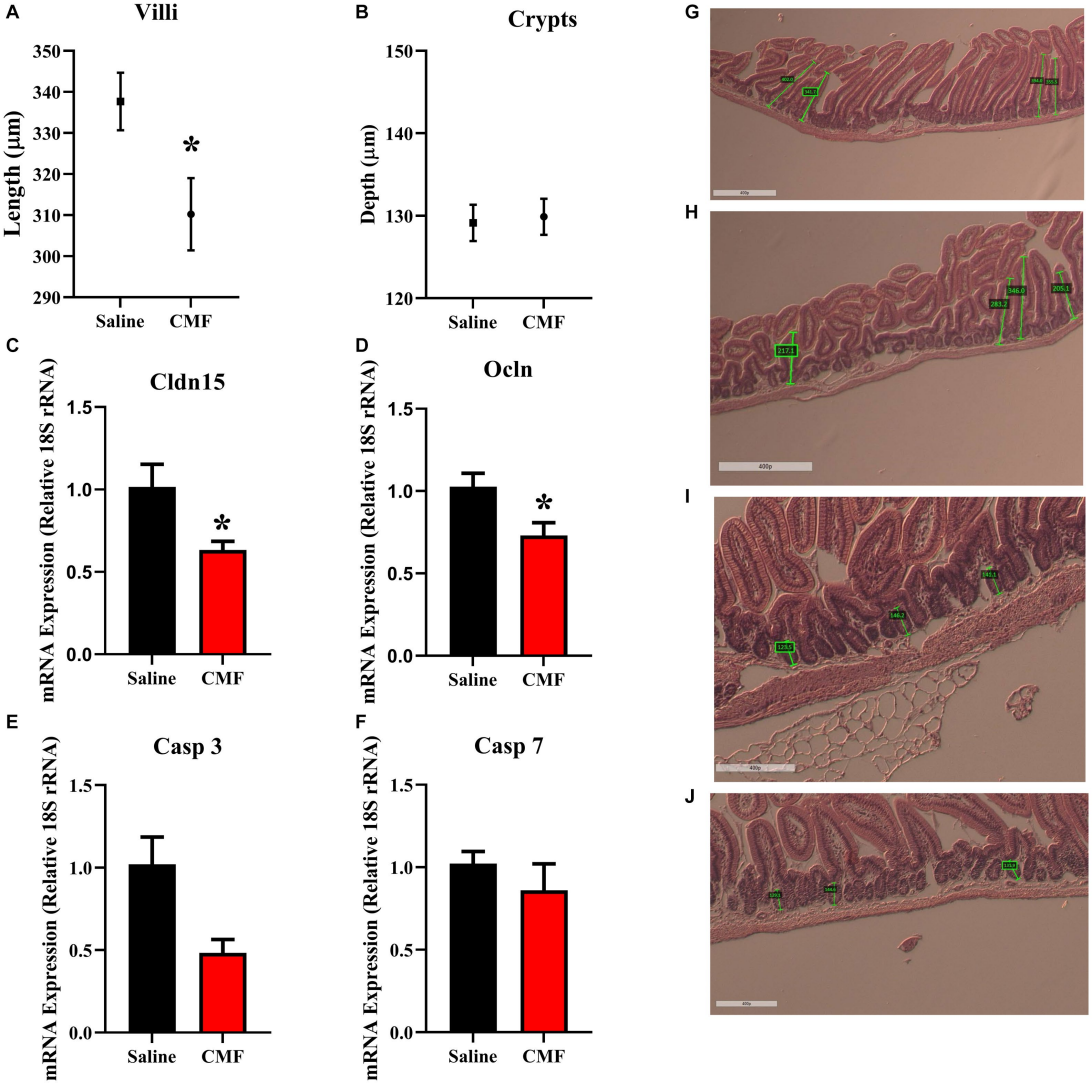
CMF induces early alterations Villi morphology but not Crypts. **(A)** The average villi length was significantly shorter when CMF were compared to Saline treated mice **(B)** CMF did not affect crypt depth (*n* = 5 per treatment). **(C,D)** shows changes in intestinal gap junction gene expression **(E,F)** no changes in Caspase expression (*n* = 8 per treatment). Representations of **(G)** saline treated and **(H)** CMF treated villi and **(I)** saline treated and **(J)** CMF treated crypts shown in histology images. *p < 0.05; †*p* < 0.01; ‡*p* < 0.001; ⁑*p* < 0.0001.

The average villi length (Figures 13A,H,G) was significantly shorter (*t* = 2.46, *p* < 0.05) when CMF (310.2 μm) were compared to Saline treated mice (337.7 μm) whereas, crypt depth (Figures 13B,I,J) was not significantly modulated (*t* = 0.24, *p* = 0.81). CMF treatment significantly decreased Claudin 15 (*t* = 3.10, *p* < 0.05, Figure 13D) and Occludin (*t* = 2.61, *p* < 0.05, Figure 13E) mRNA expression but not Caspase 3 (*t* = 2.10, *p* = 0.06; Figure 13E) or Caspase 7 (*t* = 0.91, *p* = 0.38 Figure 13F).

## 4. Discussion

The current study addresses chemotherapy-induced changes in the amygdala and hippocampus of 12-week-old female mice immediately after a 4-week cycle of CMF. We found that CMF induces depressive-like behaviors in CMF-treated mice in the FST. Also, we observed that CMF induces deficits in social novelty. A proteomic assessment of the amygdala and hippocampus suggests that dysregulation of some proteins is associated with cognitive changes. An increase in NR1, NR2A, NR2B, and GluA1 suggests glutamate excitotoxicity. Lastly, an analysis of the microbiome shows changes in alpha and beta diversity.

First, we evaluated the induction of depressive-like behaviors and emotional processing, of which the amygdala plays a role, through CMF treatment. The FST or Porsolt swim test was developed for preclinical studies for testing antidepressants and is useful in basic research related to the neurobiology and genetics of mood disorders (Can et al., 2012). In our study, we see a significant increase in immobility time as compared to saline-treated controls. Studies have shown that chemotherapy can induce depressive-like behavior and be ameliorated by an anti-inflammatory regimen (Abdelkader et al., 2017; Micov et al., 2020; Walker et al., 2020; Zhang et al., 2020) and has been shown specifically in CMF studies (Iarkov et al., 2016). In these studies, chemotherapy has been found to upregulate various cytokines, oxidative stress, glutamate receptors, immune cells, and other immune factors associated with an increased inflammatory response in the brain. These regimens act as an anti-inflammatory and assist in reducing depressive-like behaviors in chemotherapy-treated murine models.

We evaluated the chemotherapy-induced change of the hippocampus, which is known to facilitate functions for social memory. The 3-chamber social test was originally designed to test social interactions and memory (Moy et al., 2004) in mouse models for autism spectrum disorders. Mice have a natural tendency to approach and investigate novelty, much like the way a person would greet a stranger. The sociability test exploits a mouse’s tendency to prefer novelty and tests short-term memory (Yang et al., 2011). We found that mice treated with CMF were unable to show a preference for novelty over familiarity (Stranger 1 and Stranger 2) perhaps due to a deficit in social memory and being unable to recognize the novel stranger. It has been shown that chemotherapy can induce social memory changes in murine models (Whittaker et al., 2016; Zhou et al., 2016; Ntagwabira et al., 2022).

Using the IPA program, we analyzed protein expression within the amygdala and hippocampus to identify protein dysregulation that may be associated with cognitive dysfunction related to a depressive disorder that could be induced by chemotherapy treatments. With the disease and function overlay tool in IPA, we identified proteins and networks in the amygdala in the CMF-treated mice as compared to the saline-treated mice (Figure 6) are associated with damage to the nervous system and neurons, loss of neurons, primary anxiety disorder. These overlays as previous mention gives a descriptive analysis of how these proteins may be associated with the conditions in question. In this section and the following, the proteins discussed were found to be common among the disease and function overlays which include EPO, NFT3, and T3. In the network, erythropoietin (EPO) expression was predicted to be inhibited. EPO is a glycoprotein hormone excreted by the kidney in response to low oxygen levels. Many studies show EPO has neuroprotective properties and is produced in neurons, glial cells, and endothelial cells. EPO has shown neuron protection from apoptosis induced by ethanol and lithium–pilocarpine-induced status epilepticus in different parts of the brain such as the hippocampus, cerebellum, and prefrontal cortex (Kumral et al., 2005; Sözmen et al., 2012; Vittori et al., 2021). EPO has also improved cognitive function in mice with behavioral tasks such as the Morris water maze, sociability test, Y-maze, etc. (Sargin et al., 2011; Vittori et al., 2021; Garmabi et al., 2022). The inhibition of EPO suggests that there is a loss of neuroprotective function. L-triiodothyronine (T3) is a hormone produced by the thyroid. Neurotrophin-3 (NFT3/NT-3) is part of a family of neurotrophins responsible for maintaining cell survival and neuronal plasticity. An increase in neurotrophin signaling in the brain and the amygdala have been associated with fear and depressive-like behaviors (Yee et al., 2007). Supplementation of T3 has been shown to increase mitochondria activity in the amygdala and acts as an antidepressant and mood stabilizer in rats (Pinna et al., 2003).

Next, we evaluated the protein makeup of the hippocampus in the CMF-treated mice compared to saline (Figure 7). Similarly, to the amygdala, used the disease and function overlay to identify altered proteins that may be associated with neurological disorders, mild disruption of the blood–brain barrier, working memory deficit, and disinhibited behavior in the hippocampus. These overlays shared a common protein, MAPT which is predicted to be inhibited. MAPT (microtubule-associated protein tau) is a protein that plays a crucial role in maintaining the structural integrity and function of neurons. MAPT inhibition has been implicated as one of the potential mechanisms underlying chemotherapy-induced cognitive dysfunction, and it may play a role in the cognitive impairments observed in patients receiving CMF chemotherapy. The drugs in the CMF chemotherapy regimen can cause neuronal dysfunction and cognitive impairment through various mechanisms, including oxidative stress, inflammation, and disruption of cellular metabolism (Seigers and Fardell, 2011). These effects can lead to the formation of abnormal tau aggregates and inhibit MAPT function. Methotrexate has been shown to inhibit MAPT by disrupting the microtubule network in neurons (Elens et al., 2019; Rao et al., 2020; Baran et al., 2022). Methotrexate-induced inhibition of MAPT can lead to the formation of abnormal tau aggregates and contribute to the development of cognitive dysfunction (Elens et al., 2019). Cyclophosphamide and fluorouracil can also contribute to the development of cognitive dysfunction through various mechanisms that may be related to MAPT inhibition such as a decrease in myelin production (Okeda et al., 1984; Akiba et al., 1996; Han et al., 2008). A study has suggested that MAPT inhibition may also have negative effects on myelination through oligodendrocyte differentiation and formation (Seiberlich et al., 2015). Overall, MAPT inhibition is believed to be one of the contributing factors to chemobrain. This proteomics analysis is a descriptive analysis that will need further investigation but gives us insight into possible mechanisms that could be associated with cognitive dysfunction and further research would need to be done to establish a possible correlation with the gut microbiome.

Glutamate excitotoxicity is described as a mechanism of cell death triggered by excessive glutamate release from neurons and glial cells. Using PCR, we identified changes in the expression of NMDA and AMPA in the amygdala (Figure 8) and the hippocampus (Figure 9). This is regulated by major ion channel glutamate receptors, N-methyl-D-aspartate receptors (NMDARs), and α-amino-3-hydroxyl-5-methyl-4-isoxazole-propionate receptors (AMPARs), which are also responsible for sensory transmission, synaptic plasticity, learning and memory, and circuit development (Traynelis et al., 2010). In the amygdala, we see a significant expression in NR1 and NR2B. And in the hippocampus, NR2A, NR2B, and GluA1. The abundance of glutamate receptors throughout the CNS could mean that its levels need to be strictly controlled, otherwise excessive activation of glutamate receptors occurs, leading to cell swelling, apoptosis, and neuronal death. Additionally, excitotoxicity may arise as the result of abnormal functioning of glutamate receptors, such as tau-induced alterations in the phosphorylation of NMDARs even when concentrations of glutamate are normal (Ittner et al., 2010; Wang and Reddy, 2017). Hyperactivity of these receptors also is associated with behavioral changes. Increases in glutamate receptor expression levels in the basolateral amygdala resulted in interrupted excitatory synaptic transmission causing behavior deficiencies and upregulated immune response seen in these studies (Felix-Ortiz and Tye, 2014; Sharp, 2017; Hu et al., 2022) as well as the hippocampus (Carvajal et al., 2016; Gabrieli et al., 2021). It has also been suggested that NMDA has a strong association with anxiety-like behaviors in rodents in both the amygdala and hippocampus (Barkus et al., 2010; Jiang et al., 2010; Xiao et al., 2012; Lehner et al., 2017; Lorigooini et al., 2020; Wang et al., 2022). This suggests that the increase in these glutamate receptors could lead to neuronal cell death and facilitate cognitive dysfunction leading to the depressive-like behaviors and social memory impairments seen in our study.

The gut–brain axis is defined as the bidirectional communication between the gut microbiome and the brain, it plays an important role in the pathophysiology of many neuropsychiatric diseases (Carabotti et al., 2015; Appleton, 2018). Chemotherapy has also been shown to shift microbial species from commensal to pathogenic (Gori et al., 2019). The microbes in the gut are thought to be linked to the brain by producing neurotransmitters. Research has shown one of many direct relationships between microbes and xenobiotics. TIMER (Translocation, Immunomodulation, Metabolism, Enzymatic degradation, and Reduced diversity) is thought to be one of the many mechanisms behind chemotherapy modulation of the gut (Alexander et al., 2017). In studies with paclitaxel, the chemotherapy-induced significant changes in the relative abundance of microbial species during chemotherapy treatments (Loman et al., 2019). In our study, we see a visual reduction in microbial diversity in animals treated with chemotherapy as depicted in the Sankey plots. We also determined that there is a significant difference in alpha diversity in the Shannon, Faith’s, and observed features plots. PCoA plots for beta diversity also depict separation in populations between the two treatment groups. Significantly, we see changes in presence of Muribaculaceae and Romboutsia between the two treatment groups as identified by differential abundance analysis. Muribaculacease is a common bacteria found within the gut and is suggested that it is a commensal bacterium, but uncultured species are found in the presence of chemically induced inflammation (Ormerod et al., 2016). Romboutsia has been suggested to be a commensal bacterium when abundance is more relevant (Magruder et al., 2020; Zhang et al., 2022) but in chemobrain models, it has been found to take on a pathogenic phenotype (Grant et al., 2021) that is associated with inflammation. Chemotherapeutics, like 5-fluorouracil, methotrexate, irinotecan, and doxorubicin, cause weakening of the gastrointestinal lining through induced apoptosis, and these structural and functional changes allow for upregulation of the inflammatory response (Keefe et al., 2004; Sougiannis et al., 2021; Zhao et al., 2022). Chemotherapy for cancer treatment has caused disruptions in the intestine leading to a clinical diagnosis of mucositis. Clinical research on the effects of chemotherapy on the microbiome show a loss of richness and diversity after chemotherapy treatments compared to microbial conditions before the start of chemotherapy (Oh et al., 2021). Mucositis has also been reported often with chemotherapy treatment (Keefe et al., 2000; Keefe, 2004; Keefe et al., 2004). A decrease in Bacteroidetes, Bifidobacteriaceae, Clostridiaceae, Lactobacillaceae, and Muribaculaceae families have been associated with increased severity of mucositis and inflammation (van Vliet et al., 2010; Hong et al., 2019; Zhao et al., 2022) which the two bacteria species have been found within these groups. It is unclear how these to bacteria are directly associated with this model, but it is a start to a better understanding of the effects of this drug paradigm after treatments. In summary, further research would need to perform to specify the purpose of these bacteria in this chemotherapy paradigm.

The epithelium of the small intestine is organized into self-renewing crypt-villus units. Villi are finger-like protrusions of the gut wall that project into the gut lumen (Clevers, 2013). The crypt mainly functions as an architectural unit of the stem cell niche. They protect stem cells from luminal content and provide the required number of amplifying cells (Sumigray et al., 2018). Chemotherapy-induced mucositis is characterized by crypt loss, villus atrophy, loss of renewal capacity, and impairment of the gut absorptive and barrier function (Sonis et al., 1990; Sougiannis et al., 2019). Antineoplastic compounds such as 5-fluorouracil (5FU) and doxorubicin (DOX) target this otherwise healthy tissue by interrupting DNA synthesis leading to apoptotic cell death. In the current study, we found CMF treatment significantly induce the flattening of the villi but had no major effects on the crypts. These findings are in line with previous studies where cytotoxic drugs impair the turn-over of intestinal epithelia and induce flattening of the villi (Keefe et al., 2000).

To delineate the underlying mechanisms of chemotherapy-induced gut toxicity, we examined the molecular changes in key tight junction proteins Claudin-15 and occludin. Claudins are essential components of the intercellular tight junction, dysfunction may contribute to epithelial permeation and several intestinal diseases (Bertiaux-Vandaele et al., 2011). Claudins both create the paracellular barrier and determine which ions and/or molecules can selectively cross it (Tsukita et al., 2008; Amasheh et al., 2009). Occludin is a key transmembrane protein integral to tight junction integrity (McCarthy et al., 1996). CMF treatment significantly decrease Claudin 15 and Occludin expression but did not affect Caspase 3 and 7. Claudin-15 modulates small intestinal Na + permeability by forming Na + channels (Alberini et al., 2017). In addition, decreased Claudin-15 has been found in the inflamed region of human colonic biopsies of patients with ulcerative colitis, as well as in the colitis animal model (Chatterjee et al., 2021). Deleting Occludin leads to chronic inflammation and a defective epithelial barrier, which is implicated that its crucial role in tight junction stability Schulzke et al. (2005).

## 5. Conclusion

Previous studies from our laboratory showed drug combination induces cognitive decline (Anderson et al., 2020). In this study, mice were found to have decreased memory retention in behavioral testing and changes in dendritic morphology within the hippocampus for CMF-treated mice. Our study shows an increase in despair-like behaviors and social memory along with changes in protein and mRNA expression in both the amygdala and hippocampus earlier in the treatment cycle. We also see a shift in the microbial population in these treated animals post chemotherapy. In conclusion, we have shown chemotherapy induces changes in cognition while simultaneously inducing changes in microbial population. More research would need to be done to determine mechanism contribution and how specific bacteria are driving those mechanisms. Finding of this research provide evidence that cognitive changes that occur in tandem with changes in the intestine and microbiome are results of the gut-brain axis modulation. These results provide further grounds to investigate whether these initial changes after chemotherapy enable long-term changes after chemotherapy treatments. With this drug paradigm continuing to be used as an inexpensive method of treatment for breast cancer, it is important to continue to identify methods to alleviate the burdens this treatment may impose during and after treatment.

## Data availability statement

The data generated for 16ssRNA sequencing in this study can be found in the GenBank SRA under BioProject PRJNA948457; (https://www.ncbi.nlm.nih.gov/bioproject/?term=PRJNA948457). The mass spectrometry proteomics data have been deposited to the ProteomeXchange Consortium via MassIVE MSV000092957; (https://massive.ucsd.edu/ProteoSAFe/dataset.jsp?task=3aa00189aa614d7c86f8c39c1b76b49e).

## Ethics statement

The animal study was approved by Institutional Animal Care and Use Committee at the University of Arkansas for Medical Sciences. The study was conducted in accordance with the local legislation and institutional requirements.

## Author contributions

AA: conceptualization and funding. CC and TM: behavioral testing. CC and AA: statistical analyses. CC, TM, MT, DS, and PS: animal sacrifice and tissue collection. BS: PCR assay and analysis. CC, SK, and MR: QIIME 2 analysis. CC, DS, AA, and MR: writing—original draft preparation. All authors contributed to the article and approved the submitted version.
