# Supplementary material for: Physiological and cognitive changes after treatments of cyclophosphamide, methotrexate, and fluorouracil: implications of the gut microbiome and depressive-like behavior
Source: Front Neurosci. 2023 Oct 6;17:1212791. doi: 10.3389/fnins.2023.1212791 (PMC10587567; doi:10.3389/fnins.2023.1212791)
Supplement: Supplementary file 1 [file Table_1.DOCX]

| **Assay ID** | **Gene Symbol** |
| --- | --- |
| Mm00433790_m1 | NR1 (Grin1) |
| Mm00433802_m1 | NR2A (Grin2A) |
| Mm00433820_m1 | NR2B (Grin2B) |
| Mm00433753_m1 | GluA1 (Gria1) |
| Mm00442822_m1 | GluA2 (Gria2) |

**Table 1: TaqMan Assay ID** This list contains assay IDs used for PCR.

| **Gene Symbol** | **Synonym(s)** |
| --- | --- |
| ALB | Alb-1, Albumin |
| APOA1 | Alp-1, Apo1a |
| Apoc1 | apo-CI, Apo-CIB, apolipoprotein C-I |
| Apoc1 | ALPCI, Apo-CIB |
| Apoc3 | ApoC-III, apolipoprotein C3 |
| APOF | AI255964, apolipoprotein F |
| bile salt | BS |
| CLIC6 | 5730466J16RIK, AL022908 |
| CUBN | cubilin, cubilin (intrinsic factor-cobalamin receptor) |
| Ddx3x | Ddx3, DEAD-box helicase 3, X-linked |
| dimyristoylphosphatidylglycerol | 1,2-dimyristoyl-sn-glycerol-3-phosphoglycerol |
| ERK1/2 | MAPK p44/42 |
| GOT1L1 | Aspartate racemase |
| GPR119 | G protein-coupled receptor 119 |
| HDL | high-density lipoprotein |
| HDL-cholesterol | Hdl, HDL-C |
| homocysteine thiolactone | 3-aminodihydro-2(3H)-thiophenone |
| HPX | haemopexin |
| HSP90AA1 | heat shock protein 90 alpha family class A member 1 |
| ICAM1 | Melanoma Progression Associated Antigen |
| IGKV2-40 | immunoglobulin kappa variable 2-40 |
| Insulin | Ins, Ins1/2, proinsulin |
| KCTD8 | potassium channel tetramerisation domain containing 8 |
| LDL | Low density lipoprotein |
| MAPT | microtubule-associated protein tau |
| Marcks | Myristoylated alanine rich protein kinase C substrate |
| Mpo | myeloperoxidase |
| NFkB (complex) | NF-κ B, nuclear factor-κ b |
| OR2L13 | Olfactory receptor 166 |
| SAA2 | Serum amyloid A2 |
| SERPINA1 | Alpha-1-antiproteinase |
| Tcf 1/3/4 |  |
| UNC13C | Unc-13 homolog C |
| valine | 2-amino-3-methylbutanoic acid |
| ZNF558 | Zinc finger protein 558 |

**Table 2: Complete Protein List for the Hippocampus** The list was exported form the IPA program.

| **Symbol** | **Synonym(s)** |
| --- | --- |
| ALDH1A2 | aldehyde dehydrogenase 1 family member A2 |
| ARMH1 | Armadillo-like helical domain containing 1 |
| arylesterase | A-esterase, aromatic esterase |
| CA1 | Carbonic Anhydrase, Carbonic anhydrase 1 |
| CA4 | Carbonic anhydrase 4 |
| Carbonic anhydrase | Carbonate anhydrase |
| CDX Gastrointestinal Cancer Signaling Pathway |  |
| CDX2 | caudal type homeo box 2 |
| CLOCK | Circadian locomotor output cycles kaput |
| DNA (cytosine-5-)-methyltransferase | Cytosine 5-methyltransferase |
| DYNLL1 | Cytoplasmic dynein |
| EPO | epoetin |
| GATA1 | GATA binding protein 1 |
| Glutaryl-CoA Degradation |  |
| HBA1/HBA2 | Alpha-Globin |
| hydro-lyase | EctC, HPAH, hydratase |
| IHH | BDA1, HHG-2, Indian hedgehog signaling molecule |
| KCNG4 | Potassium voltage-gated channel modifier subfamily G member 4 |
| KCNK9 | Potassium channel, subfamily K, member 9 |
| L-triiodothyronine | (2S)-2-amino-3-[4-(4-hydroxy-3-iodophenoxy)-3,5-diiodophenyl]propanoic acid |
| LIN28B | Lin-28.2, lin-28 homolog B |
| LNX1 | ligand of numb-protein X 1 |
| MAFA | v-maf musculoaponeurotic fibrosarcoma oncogene family, protein A (avian) |
| MAFB | Basic domain/leucine zipper transcription factor |
| MAPK6 | Mitogen-activated protein kinase 6 |
| NR2C2 | Nuclear receptor subfamily 2 group C member 2 |
| NTF3 | Neurotrophin 3, NGF-2, NT-3 |
| PLCB1 | Phosphoinositidase C |
| SOST | CDD, DAND6, SCLEROSTIN, SOST1, VBCH |
| TFCP2 | Alpha-globin transcription factor cp2, transcription factor CP2, UBP-1 |
| Tryptophan Degradation III (Eukaryotic) |  |
| TTC23 | tetratricopeptide repeat domain 23 |
| UPF3A | UPF3A regulator of nonsense mediated mRNA decay, UPF3A |

**Table 3: Complete Protein List for the Amygdala** The list was exported form the IPA program.
